# Dione: An OWL representation of ICD-10-CM for classifying patients’ diseases

**DOI:** 10.1186/s13326-016-0105-x

**Published:** 2016-10-13

**Authors:** María del Mar Roldán-García, María Jesús García-Godoy, José F. Aldana-Montes

**Affiliations:** 1 Ada Byron Research Center, University of Malaga, Ampliación del Campus de Teatinos, Málaga, Spain; 2 IBIMA Instituto de Investigación Biomédica de Málaga, University of Malaga, Málaga, Spain

**Keywords:** ICD-10-CM, SNOMED CT, Ontologies, Automatic classification

## Abstract

**Background:**

Systematized Nomenclature of Medicine - Clinical Terms (SNOMED CT) has been designed as standard clinical terminology for annotating Electronic Health Records (EHRs). EHRs textual information is used to classify patients’ diseases into an International Classification of Diseases, Tenth Revision, Clinical Modification (ICD-10-CM) category (usually by an expert). Improving the accuracy of classification is the main purpose of using ontologies and OWL representations at the core of classification systems. In the last few years some ontologies and OWL representations for representing ICD-10-CM categories have been developed. However, they were not designed to be the basis for an automatic classification tool nor do they model ICD-10-CM inclusion terms as Web Ontology Language (OWL) axioms, which enables automatic classification. In this context we have developed Dione, an OWL representation of ICD-10-CM.

**Results:**

Dione is the first OWL representation of ICD-10-CM, which is logically consistent, whose axioms define the ICD-10-CM inclusion terms by means of a methodology based on SNOMED CT/ICD-10-CM mappings. The ICD-10-CM exclusions are handled with these mappings. Dione currently contains 391,669 classes, 391,720 entity annotation axioms and 11,795 owl:equivalentClass axioms which have been constructed using 104,646 relationships extracted from the SNOMED CT/ICD-10-CM and BioPortal mappings included in Dione using the owl:intersectionOf and the owl:someValuesFrom statements. The resulting OWL representation has been classified and its consistency tested with the ELK reasoner. We have also taken three clinical records from the Virgen de la Victoria Hospital (Málaga, Spain) which have been manually annotated using SNOMED CT. These annotations have been included as instances to be classified by the reasoner. The classified instances show that Dione could be a promising ICD-10-CM OWL representation to support the classification of patients’ diseases.

**Conclusions:**

Dione is a first step towards the automatic classification of patients’ diseases by using SNOMED CT annotations embedded in Electronic Health Records (EHRs). The purpose of Dione is to standardise and formalise a medical terminology, thereby enabling new kinds of tools and new sets of functionalities to be developed. This in turn assists health specialists by providing classified information from EHRs and enables the automatic annotation of patients’ diseases with ICD-10-CM codes.

**Electronic supplementary material:**

The online version of this article (doi:10.1186/s13326-016-0105-x) contains supplementary material, which is available to authorized users.

## Introduction

The International Classification of Diseases, 10th Revision (ICD-10) [1] is a standard diagnostic tool for health management, epidemiology and clinical purposes. ICD-10 comprises Chapters I to XXII which cover diseases, a variety of signs and symptoms, abnormal findings, complaints, social circumstances and external causes of injuries and diseases. ICD-10-CM corresponds to the tenth version, clinical modifications, which is the current ICD version. This medical classification standard, maintained and published by the World Health Organisation (WHO) is used to classify diseases and health problems that have been recorded on death certificates and in other records. The accuracy of this classification is a very important issue because it is used, for example, to set capitation rates and allocate resources to medical centers. It is also used by medical and health services researchers to determine the case fatality and morbidity rates. Furthermore, ICD-10-CM has been mandatory in the U.S. and therefore in regular and routine use since October 1, 2015.

The concept of ontologies has been widely used in numerous real-word applications domains from Health Care and Life Science to Finance and Government. The majority of current ontologies are expressed in the well-known Web Ontology Language (OWL) [2]. Semantic Reasoners such as Pellet [3], ELK [4], KAON2 [5] and RacerPro [6] are all widely used to develop ontology-based automatic classification systems. Improving the accuracy of classification is the main purpose of using ontologies and OWL representations as the basis for a classification system. The literature review refers to several OWL ontologies for representing ICD-10-CM categories that have been developed. However, they were never intended to be the basis for an automatic classification tool nor do they model ICD-10-CM inclusion terms as OWL axioms, which enables this type of automatic classification.

**Fig. 1:**
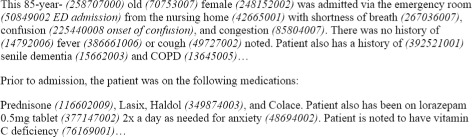
SNOMED CT codes embedded in an Electronic Medical Record [44]. EHRs are annotated using SNOMED CT concepts (in parentheses). For example, the SNOMED CT concepts 248152002 and 267036007 correspond to *Female* and *Shortness of Breath*, respectively

Systematized Nomenclature of Medicine - Clinical Terms (SNOMED CT) [7] is a broad clinical terminology which covers a wide range of disciplines, clinical specialties and requirements. One of the main aims of SNOMED CT is for it to be used as the standard terminology in Electronic Health Records (EHRs) systems. The use of SNOMED CT enables providers and EHR to use a common language. Therefore, EHRs are annotated using several SNOMED CT concepts and relationships between SNOMED CT concepts, which codify patients’ information such as previous diseases, affected part of the body, and symptoms. Figure 1 shows an excerpt from an EHR where SNOMED CT codes are embedded.

SNOMED CT/ICD-10-CM alignments (mappings) have been established and described by Unified Medical Language System (UMLS) [8]. These mappings have a cardinality of many to many. This means that one SNOMED CT concept can be mapped with many ICD-10-CM target categories and vice versa. When a SNOMED CT concept is not mapped to an ICD-10-CM category it means that it is not classifiable or is awaiting editorial review. One or more SNOMED CT source concepts can also be mapped with the same ICD-10-CM target category. In addition to these mappings provided by UMLS, BioPortal’s users have also defined SNOMED CT/ICD-10-CM mappings [9]. In this case, the Bioportal’s mappings have the same cardinality as the mappings provided by UMLS.

The current situation is that EHRs are annotated using SNOMED CT concepts and textual information from these records is then used to classify the patients’ diseases into an ICD-10-CM category (usually by an expert). Interestingly, SNOMED CT/ICD-10-CM mappings can be exploited to define the ICD-10-CM inclusion terms based on the SNOMED standard definition of patients’ medical evidence, affected part of the body and symptoms and their relationships, connecting the standard annotations in EHRs with an ICD-10-CM category.

The main hypothesis of the work presented here is: **(H1) It is possible to code, as OWL axioms, the ICD-10-CM inclusion terms obtained from SNOMED CT/ICD-10-CM mappings and use these OWL axioms to build an OWL representation of the ICD-10-CM diseases**. As a result **(H2) we obtain a useful OWL representation, which can be used as the basis for a semantic classification system**. This system will then use the set of SNOMED CT concepts and relationships between SNOMED CT concepts, taken from EHRs, as input to automatically classify patients’ diseases into an ICD-10-CM category. The objective of using this OWL representation is to improve the accuracy of the manual annotation. The accuracy of the classification is particularly relevant at a time when diagnoses codes, such as the ICD-10-CM codes, can significantly affect the total funding that a hospital may receive for patients admitted [10]. For example, in the United States, diagnosis-related groups (DRGs) based on ICD codes are the basis for hospital reimbursement for acute-care stays of Medicare beneficiaries [11]. Another fact is that health services researchers use the ICD codes to study risk-adjusted, cross-sectional, and temporal variations in access to care, quality of care, costs of care, and effectiveness of care [12].

This has motivated us to develop an OWL representation to help find an automated approach to classify patients’ diseases in a medical context. Inclusion terms for each ICD-10-CM category are formalised as OWL axioms by exploiting SNOMED CT/ICD-10-CM mappings. These mappings allow SNOMED CT concepts and relationships to be used to define the inclusion terms. The resulting OWL representation called Dione^1^, which includes the ICD-10-CM Chapters I to XIV, is as complete as the available SNOMED CT/ICD-10-CM mappings allow. The exclusions proposed by ICD-10-CM are already handled by the mappings.

The main contributions of this paper are summarised as follows:

- We have defined an algorithm which, starting from an ICD-10-CM category code, is able to obtain its corresponding SNOMED CT concept and all its relationships.
- The relationships obtained are considered to be the inclusion terms for the ICD-10 category. Therefore, we have also defined an algorithm for translating these relationships to OWL axioms.
- To test H1 we have developed Dione, an OWL representation of ICD-10-CM, specifically designed to classified patients’ diseases by exploiting OWL’s (Description Logic) reasoning capabilities. Dione represents the ICD-10-CM categories as classes and the inclusion terms as OWL axioms related to the class by means of the owl:equivalentClass statement. Classes representing ICD-10-CM categories as well as classes representing SNOMED CT concepts are organised as several hierarchies.
- To test H2, Dione’s consistency has been checked and information from real clinical records has been classified to show Dione’s applicability through three clinical use cases from the Virgen de la Victoria Hospital (Málaga, Spain).

## Background

There have been some attempts made to construct OWL models using biomedical classifications like SNOMED CT and ICD-10. For ICD, the work developed by [13] was the first attempt to model the ICD-9 ontology, an older version of the current ICD-10. In [14], the authors proposed the first formal representation of the ICD-10 based on three logical layers of the GALEN Core Reference Model (CRM) terminology system [15]. They used a description logic-like language called GRAIL [16] which allows classes to be inferred with the semantics of role propagation and links a more detailed description of a diagnosis to a more abstract class. The ICD-10 ontology presented in [14] contains only ICD-10 categories and their definitions. The hierarchical relationship of the ICD-10 is not represented and the ICD-10 category definitions are limited to three concepts defined by a multi-axial conceptual system that includes the anatomy, the morphology and the etiology. However, it has to be said that the methodology adopted by the authors to formalise the ICD-10 has some limitations: first, only two ICD-10 chapters are represented; second, not all the ICD terms are represented using GALEN and finally, the ontology was not loaded into an OWL reasoner and therefore, the formal consistency was neither checked nor classified. Given these problems, the authors presented a DOLCE-based formal representation [17]. DOLCE is a descriptive upper-level ontology designed for ontology cleaning and interoperability. In this formal representation of the ICD-10, anatomical entities were taken from the Foundational Model of Anatomy (FMA) [18], morphological abnormalities and procedures were taken from SNOMED CT, the organisms used were from the biological taxonomy and the chemical objects were taken from the International Union of Pure and Applied Chemistry nomenclature (IUPAC). Despite these improvements over the previous version of the GALEN-based ICD-10 representation, some problems have yet to be solved. For example, *not elsewhere classified* diseases are modeled as logical exclusions of *elsewhere classified* ICD categories from the appropriate parent concepts. This solution does not provide any information for a system which aims to automatically classify a patient’s disease *d*. The doctor should assert or the system should infer that *d* is an instance of the negation of a class. Due to the OWA (Open World Assumption) semantics of OWL, if *d* is not an instance of class C, the reasoner cannot infer that *d* is an instance of ¬C. Furthermore, the ontology has not been checked or classified by a reasoner.

The last approach to represent ICD-10 in OWL was developed in [19]. In this study, an ontology was created based on two super-classes, the icd10:Entry and the icd10:Modifier which contain ICD-10 codes from the WHO and the German Institute for Medical Documentation and Information (German: Deutsches Institut für Medizinische Dokumentation und Information) [20], respectively. The general structure of the ICD-10 ontology includes Chapters I to XXI; classes are represented by an URL which consists of a name space and the ICD-10 code name, and their relations are established with owl:subClassOf axioms. The ICD-10 exclusions are handled with the owl:disjointWith axiom. This approach does not provide information as to in which class the diseases should be classified. If the same disease is classified in both classes, the reasoner infers that the ontology is inconsistent, but is unable to distinguish the correct class. Furthermore, to solve the problem of exclusions that are shared with multiple exclusions, the authors proposed the inclusion of icd10:hasExcludes that links to a icd10:ICDdescription (with a rdf:type and rdf:label predicates) which has an icd10:concernsClass property. As icd10:concernClass can involve other ICD-10 categories, the ontology requires an OWL-full expressivity. The inclusions are modelled in the same way as the exclusions. The OWL-full properties presented in the ontology invalidate it for use by reasoners and thus, it is not possible to check the ontology’s consistency or classify it.

According to the literature review, there have been several attempts to model ICD-10 in OWL. However, these studies have various weaknesses which can be summarised as follows: 1) some difficulties in correctly handling inclusions and exclusions in an OWL representation of ICD-10; 2) the lack of a validation process using an OWL reasoner to check the consistency of the ontology. This step is very important in ontology development and testing [21] because an ontology can be used by OWL reasoners without human supervision. If an ontology is inconsistent, the reasoning may lead to erroneous conclusions; 3) no application of these OWL representations to real clinical use cases to show how they can support a clinician in decision making and 4) the reviewed work uses ICD-9 and/or ICD-10. In this paper, we have worked with ICD-10-CM. Although the intention is to replace ICD-9-CM with ICD-10-CM, it has been reported in [22] that for reasons such as the complexity of ICD-10-CM and the costs of migrating from one system to the other would explain why the ICD-9-CM version is still in use [22]. The Center for Disease Control and Prevention (CDC) encourages the use of the ICD-10-CM version because of the improvements that it has over ICD-9-CM and ICD-10 [23]. These improvements include the addition of information relevant to ambulatory and managed care encounters; expanded injury codes; the creation of combination diagnosis and symptom codes to reduce the number of codes needed to fully describe a condition; the addition of sixth and seventh characters; incorporation of common fourth and fifth digit subclassifications; laterality and greater specificity in code assignment [23].

## Methods

### Formalising the ICD-10-CM categories in OWL

For the construction of Dione, we focused on three basic issues that are critical when modelling an ontology or an OWL representation, and are specified in the “Ontology 101 development process” methodology [24]. First, a selection of the concepts used to cover the objectives to be accomplished in the health domain; second, the organisation of all concepts in a hierarchy and third, a semantic formalisation of these concepts using a knowledge representation language such as the description logic (DL) formalism.

In order to select the terms for each concept, an XML file containing the ICD-10-CM categories in the English version was downloaded from the Centers for Disease Control and Prevention (CDC) website that stores all the ICD versions [25]. ICD-10-CM consists of “chapters” that are sub-divided into homogeneous blocks of three-character categories (a capital letter and two arabic numerals). These categories are sub-divided by means of four-character categories (a capital letter and three arabic numerals) and these are further divided into five-character categories. The file with the ICD-10-CM categories was parsed to output a tree with parent and child nodes (Additional file 1). The upper-level and lower-level nodes of the tree generated from the XML file correspond to the ICD-10-CM upper and lower levels, which involve blocks of three-, four- and five-character categories. For the semantic formalisation, the hierarchy tree from the output file, which includes Chapters I to XIV, was encoded in OWL using the OWL API library [26]. Using the information from ICD-10-CM about blocks and their categories, the OWL hierarchy was modelled establishing the *Diseases* category as super-class because Chapters I to XIV, are related to diseases. All Dione classes are identified by an URI, which consists of a namespace and a special term which corresponds to the name of each ICD-10-CM category code. The *Diseases* category includes the following ICD-10-CM categories: A00-B99 (*Certain infectious and parasitic diseases*), C00-D49 (*Neoplasms*), D50-D89 (*Diseases of the blood and blood-forming organs and certain disorders involving the immune mechanism*), E00-E89 (*Endocrine, nutritional and metabolic diseases*), F01-F99 (*Mental, Behavioural and Neurodevelopmental disorders*), G00-G99 (*Diseases of the nervous system*), H00-H59 (*Diseases of the eye and adnexa*), H60-H95 (*Diseases of the ear and mastoid process), I00-I99 (Diseases of the circulatory system*), J00-J99 (*Diseases of the respiratory system), K00-K95 (Diseases of the digestive system*), L00-L99 (*Diseases of the skin and subcutaneous tissue*), M00-M99 (*Diseases of the musculoskeletal system and connective tissue*) and N00-N99 (*Diseases of the genitourinary system*).

**Fig. 2:**
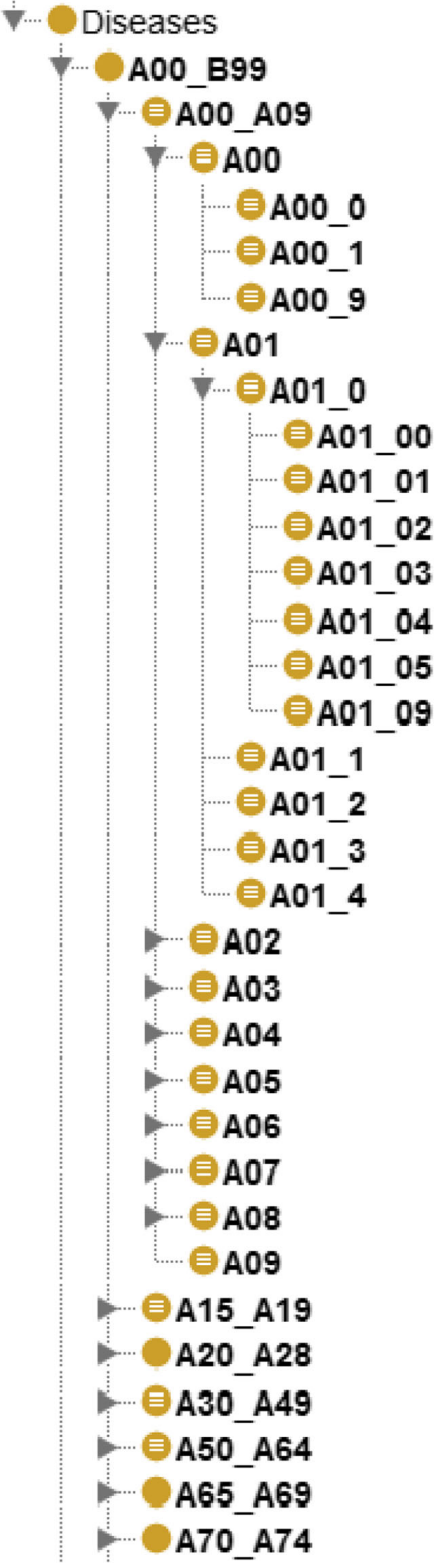
Part of the A00_B99 category hierarchy included in the *Diseases* category displayed on the Protegé software interface. A00_B99 is a subclass of the main class *Diseases*. According to the ICD-10-CM structure, A00_A09 is a subclass of A00_B99 and A00_0, A00_1 and A00_9 are subclasses of A00_A09. According to the semantics of OWL, if x is an instance of A00_9, it is also an instance of A00_B99 and therefore, it is also an instance of A00_A09

In this hierarchy, the *Diseases* class includes the ICD-10-CM disease classification, the classes inside each disease category are related to their upper-level category using the is-a relationship; these relationships are formalised in Dione with the *owl:subClassOf* axiom. Therefore, a branch of the disease ICD-10-CM hierarchy was created (Additional file 2). Figure 2 shows part of the Diseases hierarchy, specifically the A00-B99 (*Certain infectious and parasitic diseases*) branch and its subclasses. For example, A00-A09 (*Intestinal infectious diseases*) has as subclasses A00.0 (*Cholera due to Vibrio cholerae 01, biovar cholerae*), A00.1 (*Cholera due to Vibrio cholerae 01, biovar eltor*) and A00.9 (*Cholera, unspecified*). An English label with the name of each category was included for each class of the ICD-10-CM disease classification hierarchy, using the rdf:label statement. Once the Dione hierarchy had been constructed, the next step consisted in including the *owl:equivalentClass* axioms for each Dione class to model the ICD-10-CM inclusion terms. Therefore, in order to complete the semantic formalisation of the concepts of the OWL hierarchy, the SNOMED CT/ICD-10-CM mappings were used to construct the *owl:equivalentClass* axioms for each class. Where an SNOMED CT/ICD-10-CM mapping was not available, we completed Dione with the inferred mappings provided by the BioPortal website. These steps are fully explained in the following sections.

### Inclusion of the OWL axioms

The files related to SNOMED CT were downloaded from the National Institutes of Health (NIH) webpage [27]. The SNOMED CT concepts, the descriptions, the is-a relationships and the other relationships that represent other concept associations were stored in an Oracle 11g Database. The mapping files between ICD-10-CM and SNOMED CT (also known as “the map”) were downloaded from UMLS [8]. The SNOMED CT/ICD-10-CM mappings were also extracted and stored in the database. To generate the inclusion terms to be included as *owl:equivalentClass* axioms for each OWL class of the Dione hierarchy, an automatic process was implemented (Additional file 3) based on the SNOMED CT relationships that provide information about ICD-10-CM inclusion terms (see Table 2), namely, the SNOMED CT relationships that we found in EHRs which provided information for classifying patients’ diseases. Therefore, relationships representing historical attributes such as “maybe a” (ambiguous concept), “was a” (erroneous concept), “moved to” (moved to elsewhere concept), and other relationships with qualifier values such as “Episodicities” etc. have not been considered. In total, there are 12 SNOMED CT relationships that are used to define the ICD-10-CM categories. For example, the SNOMED CT causative-agent relationship, which identifies the direct causative agent of a disease which can be an organism, substance or physical force, that is represented by the caused-by-agent Dione object property. Another example is the associated-morphology relationship, which specifies the changes that are seen at the cellular level or in tissues, caused by a disease, that is represented by has-Associated-Morphology Dione object property. Table 1 shows materialised examples of such relationships.

**Table 1:** SNOMED relationships examples. Examples of SNOMED CT concept related to SNOMED CT through causative-agent and associated-morphology relationships

SNOMED CT concept	SNOMED CT relationship	SNOMED CT concept
Cholera-non-01 group vibrio, disorder	**causative-agent**	Vibrio cholerae, non-O1, an organism
Bullous pyoderma, disorder	**associated-morphology**	Chronic superficial ulcer, a morphologic abnormality

Bold data are SNOMED relationship

Once the Dione properties had been identified, the relationships between two SNOMED CT concepts (one of which maps to the ICD-10-CM code included in Dione ICD-10-CM hierarchy) were extracted and modelled by means of the *owl:equivalentClass* restriction of Dione classes and *owl:intersectionOf* statement in order to model the ICD-10-CM inclusion terms. The ICD-10-CM category was defined with the following properties: the SNOMED CT relationship (the object property in Dione), the *owl:someValuesFrom* restriction and the SNOMED CT concept. In order to illustrate this, a good use case is the I10 (Essential primary hypertension) ICD-10-CM category:

I10 (Essential primary hypertension) is mapped to these two SNOMED CT concepts:

- “Hypertensive episode (disorder)” (62275004)
- “Complication of systemic hypertensive disorder (disorder)” (449759005)

According to the ICD-10-CM guidelines, the I10 category (*Essential primary hypertension*) has the inclusion terms “High Blood pressure” and “Hypertension”, which could be considered as a set of symptoms from a given patient. The SNOMED CT structure indicates that the SNOMED CT concept “Hypertensive episode (disorder)” (62275004) is related to the concept “Finding of increased blood pressure (finding)” (24184005) by the relationship “Has definitional manifestation” (363705008). In the same way, the SNOMED-CT concept “Complication of systemic hypertensive disorder (disorder)” (449759005) is related to the concept “Hypertensive disorder, systemic arterial disorder” (38341003) by the relationship “Associated with” (47429007). The SNOMED CT concepts “Finding of increased blood pressure (finding)” (24184005) and “Hypertensive disorder, systemic arterial disorder” (38341003) are equal to “High Blood pressure” and “Hypertension”, respectively. These two inclusion terms have been modelled in Dione, by means of the *owl:someValuesFrom* statement (∃), as follows:

- ∃ hasDefinitionalManifestation.24184005^2^
- ∃ associatedWith.38341003.

**Fig. 3:**
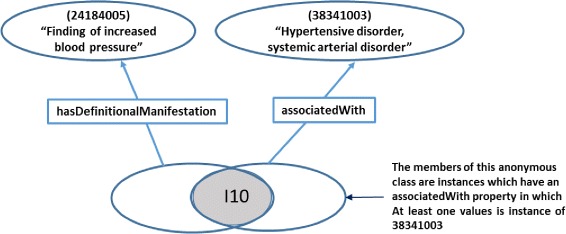
OWL definition of the Class I10. ICD-10-CM category I10 inclusion terms are modelled as *owl:someValuesFrom* statement (∃) and their intersection are defined as equivalent of the class I10 by means of the *owl:equivalentClass* (⊓) axiom. The Description Logic syntax of OWL is used

To associate the inclusion terms in a conjunctive form, the *owl:intersectionOf* (⊓) statement was used. Finally, the conjunction was associated with the class I10 through an *owl:equivalentClass* (≡) axiom. Therefore, the definition of the I10 class including the inclusion terms is the following (Fig. 3 also shows it graphically):

- I10 ≡∃ hasDefinitionalManifestation.24184005 ⊓ ∃ associatedWith.38341003

**Fig. 4:**
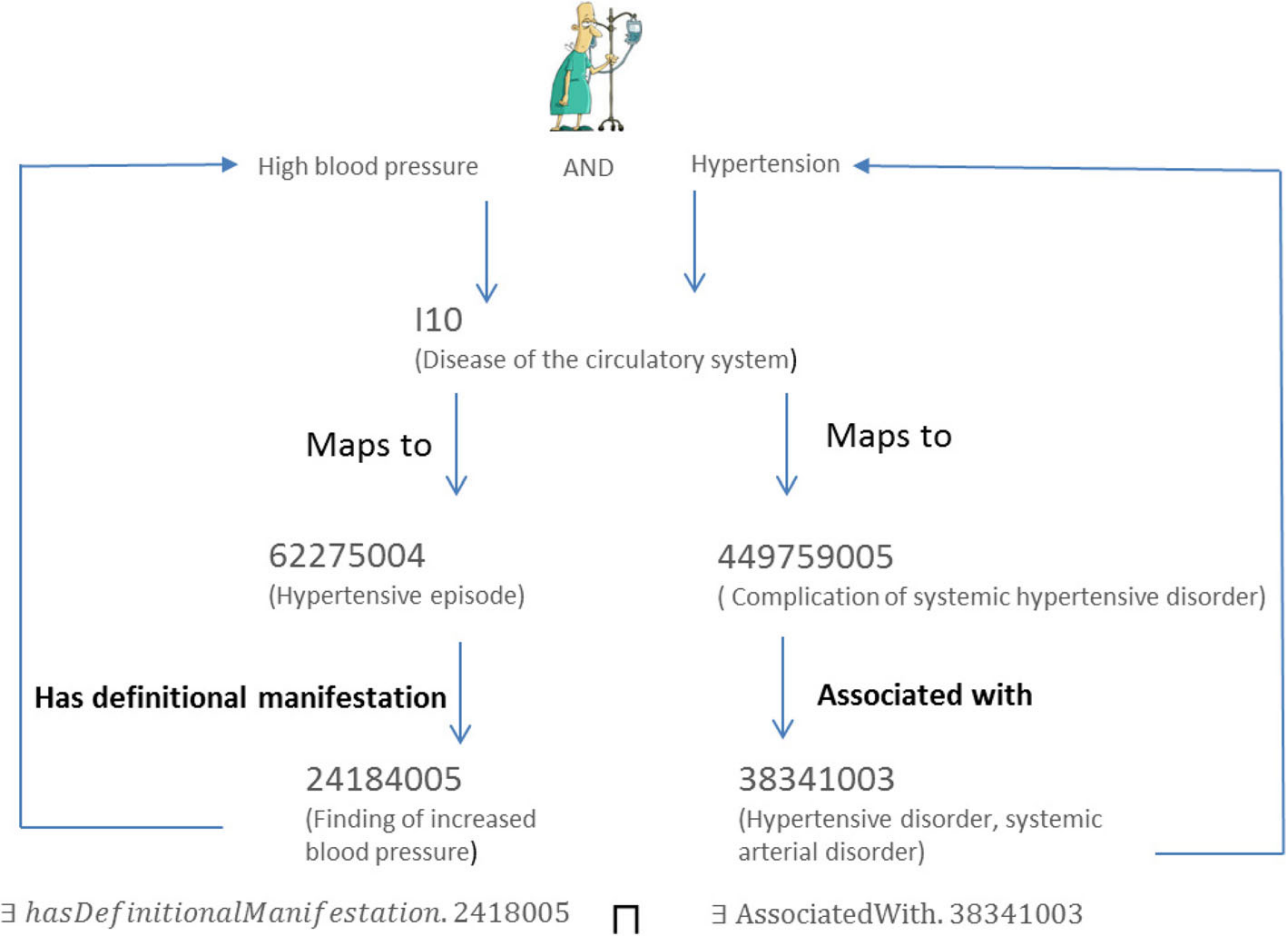
Description of the process to model I10 inclusion terms in Dione. From the SNOMED CT/ICD-10-CM mappings and by exploiting the SNOMED CT relationships the ICD-10-CM inclusion terms are modelled. This figure shows there exists a SNOMED CT/ICD-10-CM mapping for each ICD-10-CM inclusion term

Figure 4 describes how the inclusion terms of I10 (Essential primary hypertension) have been modelled in Dione. It is also worth noting that some SNOMED CT relationship names to define the Dione properties were changed to avoid any ambiguity in Dione. For example, the “Has definitional manifestation” relationship was renamed as *hasDefinitionalManifestation* object property to avoid gaps between words as displayed in the first and second columns of Table 2.

**Table 2:** SNOMED CT relationships that were used to define the Dione classes

Dione object property	SNOMED CT relationship	Description	Number of uses in Dione
Affects	Finding site	The part of the body affected by a condition	10,812
After-Of	After	Represents a sequence of events where a clinical finding occurs after another	491
Associated-With	Associated with	Represents a clinically relevant association between concepts without either asserting or excluding a causal or sequential relationship between the two	469
Caused-By-Agent	Causative agent	Identifies the direct causative agent of a disease (e.g., an organism)	1,701
Due-To	Due to	Relates a clinical finding directly to a cause such as another clinical finding or a procedure	504
Has-Associated-Finding	Associated finding	Links concepts in the situation with explicit context hierarchy to their related clinical finding	66
Has-Associated-Morphology	Associated morphology	Specifies the morphologic changes seen at the tissue or cellular level that are characteristic features of a disease	7,634
Has-Definitional-Manifestation	Has definitional manifestation	Links disorders to the manifestations (observations) that define them	818
Has-Occurrence	Occurrence	Refers to a specific period of life during which a condition first presents	520
Has-Pathological-Process	Pathological process	Provides information about the underlying pathological process for a disorder, but only when the results of that process are not structural and cannot be represented by the associated morphology relationship	1,867
Interprets	Interprets	Refers to the entity being evaluated or interpreted, when an evaluation, interpretation or judgment is intrinsic to the meaning of a concept	425
IsPartOf	Part of	Represents a sequence of events where a clinical finding occurs after another clinical finding or a procedure	11

The first and second columns include the selected SNOMED CT relationships with their Dione and original names. The third column is a description obtained from the SNOMED CT User guide [45] and the fourth column includes the number of times that these relationships were used in Dione

**Fig. 5:**
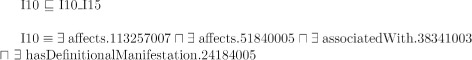
Complete definition of the class I10. In addition to the SNOMED CT/ICD-10-CM mappings representing ICD-10-CM inclusion terms we model the rest of mappings as *owl:someValuesFrom* statement (∃) in order to complete the definition of the class. ∃ affects.113257007 models that I10 affects 113257007 (Structure of cardiovascular system), ∃ affects.51840005 models that I10 affects 51840005 (Systemic circulatory system structure), ∃ associatedWith.38341003 models that I10 is associated with 38341003 (Hypertensive disorder, systemic arterial) and ∃ hasDefinitionalManifestation.24184005 models that I10 has definitional manifestation 24184005 (Finding of increased blood pressure)

The rest of the relationships of the SNOMED CT concepts mapped with I10 (Essential primary hypertension) were also included as OWL axioms to complete the definition of the class (see Fig. 5).

**Fig. 6:**
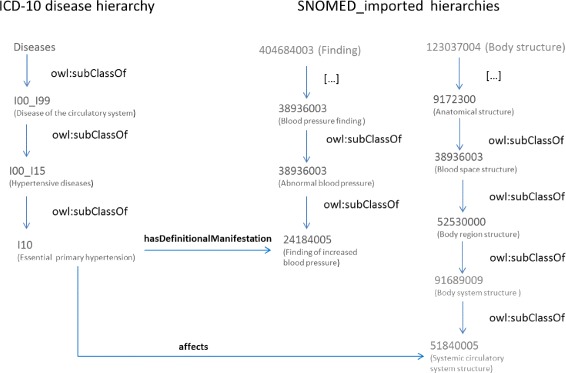
Structure of the ICD-10-CM disease hierarchy and the *owl:equivalenClass* axioms identified and included. The range of the properties in Dione corresponds to the SNOMED CT concepts imported in the SNOMED CT hierarchy

Dione is composed of several branches: 1) the ICD-10-CM disease hierarchy, which comprises Chapters I and XIV, including the concepts that are the domain of the relationships included and 2) the SNOMED CT imported concept hierarchies (and their annotations in rdf:label), which comprise those SNOMED CT concepts taken from the relationships whose ranges include these concepts. These imported concept hierarchies were created in OWL using the is-a relationship, as described in *Phase I* (Additional file 4). Like the previous example of how the inclusions are modelled for I10 class, the I10 has *associatedWith*, *hasDefinitionalManifestation* and *affects* properties identified in the *owl:equivalentClass* restriction. These properties have a range that corresponds to a SNOMED CT concept. For example, *affects* and *hasDefinitionalManifestation* have SNOMED CT concepts such as “Systemic circulatory system structure (body structure)” and “Finding of increased blood pressure (finding)” as range, respectively. These concepts are included in the Dione SNOMED CT imported hierarchy as shown in Fig. 6.

### Completion with BioPortal mappings

Once Dione had been developed, we have determined the number of classes defined with the relationships from the SNOMED CT/ICD-10-CM mappings provided by UMLS (Additional file 5). Those classes that did not involve either an inherited or non-inherited axiom (in the case of a superclass of the ICD-10-CM disease branch of Dione) were defined using the SNOMED CT/ICD-10-CM mappings from the BioPortal website [9]. To do this, the new mappings between the SNOMED CT concepts and the ICD-10-CM categories were extracted from the BioPortal API [28] and inferred to avoid duplicate SNOMED CT/ICD-10-CM mappings from NIH. Following the methodology described in the previous subsections, the new mappings were stored in the database. The new OWL statements for the inclusion terms of the ICD-10-CM categories without any axioms were generated and included in Dione. Thus, we obtained the most complete Dione version possible with the resources available, given that some classes were not defined (statistics are presented in the “Results” section).

*Completing the definition of Dione classes*

As we have mentioned, we could not find SNOMED CT/ICD-10-CM mappings for all ICD-10-CM categories. This means that we could not include OWL statements that model inclusion terms, for all Dione classes. In some cases where we did find a mapping, the SNOMED CT concept which the ICD-10-CM category was mapped to did not have relationships that could be translated into OWL statements.

This incompleteness of Dione means that a patient’s disease can be classified into ICD-10-CM categories belonging to different chapters. In order to partially solve this problem and taking into account that the main objective of Dione is to classify patients’ diseases using the SNOMED CT annotations embedded in EHRs, we have implemented a first process to include the axioms defining a class in the definition of its subclasses (Additional file 6). Then we have completed a second process to include the owl:someValuesFrom statement from the definition of sibling subclasses in the definition of their superclass (Additional file 7). Before this process, Dione defined:

- K58 (Irritable bowel syndrome) ≡∃ affects.113276009 (Intestinal structure)
- E73_0 (Congenital lactase deficiency) ≡∃ affects.113276009 (Intestinal structure) ⊓ ∃ hasOccurrence.255399007 (Congenital)

Therefore, if a patient’s disease was classified in E73_0 (Congenital lactase deficiency), it was also classified in K58 (Irritable bowel syndrome), because E73_0 was a subclass of k58. The problem here was that we found a mapping but we could not find more OWL statements to define K58 from SNOMED CT relationships. However, we did observe that K58 has two subclasses (K58_0 and K58_9) and both subclasses share ∃ affects.71854001 (Colon structure) in their definitions. Therefore, we were able to add this OWL statement to K58 and now K58 is defined as:

- K58 (Irritable bowel syndrome) ≡
- ∃affects.113276009 (Intestinal structure) ⊓
- ∃affects.71854001 (Colon structure)

With this new definition E73_0 is not a subclass of K58.

### Dione consistency and classification

For Dione classification, we used the ELK reasoner with the OWL API (Additional file 8). After some attempts to apply Dione classification with reasoner systems such as Fact++ [29], Hermit [30], Pellet [3], TrOWL [31], RacerPro [6] and CEL [32], it was found that the ELK reasoner [4] was the only reasoner able to classify Dione while simultaneously checking that Dione was consistent. Fact++, Pellet, RacerPro and CEL failed due to an out-of-memory error (heap space set to 12 GB). TrOWL and Hermit failed due to a timeout after 48 h. The experiments were performed on a PC Intel(R) Core (TM) i7-2600 CPU with 3.39 GHz and 16 GB of RAM and took 2781 s.

## Results and discussion

### Level of completion of Dione

Dione has been built based on the ICD-10-CM terms (2014 release) provided by the CDC [25] and SNOMED CT terms from UMLS (March 2013 release). Dione contains 391,669 classes, 391,720 entity annotation axioms and 19,797 *owl:equivalentClass* axioms which were constructed with 104,646 relationships extracted from the SNOMED CT/ICD-10-CM and Bioportal mappings and included in Dione using the *owl:intersectionOf* and the *owl:someValuesFrom* constructs.

**Fig. 7:**
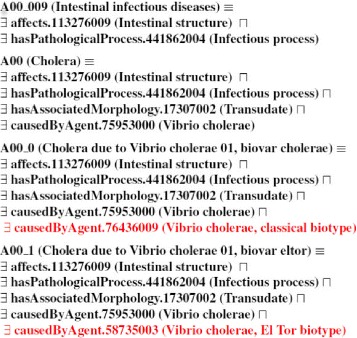
Dione classes and subclasses modeling Cholera. This is an extract of Dione which shows how Dione looks when classes have complete definitions. The definition of A00_0 and A00_1 includes an OWL statement to distinguish between the different types of Cholera

The current version of Dione has 21,616 classes for modelling ICD-10-CM categories. After using the SNOMED CT/ICD-10-CM mappings from UMLS metathesaurus, the percentage of classes with axioms was 93 %. So, to provide a more complete version of Dione, we extracted a set of ICD-10-CM/SNOMED CT mappings from BioPortal. These BioPortal mappings include one-to-one mappings and one-to-many mappings. An example of the second case is the ICD-10-CM category A93.8 (“Other specified arthropod-borne viral fevers”) which is mapped to three SNOMED CT concepts. This concept points to the SNOMED CT concept “[X] Other specified arthropod-borne viral fevers”, “Piry virus disease” and “[X] Other specified viral hemorrhagic fevers”. The relationships from these SNOMED CT concepts for both types of mappings were extracted and included in Dione to define the ICD-10-CM categories. Therefore, with the new mappings from BioPortal included in Dione as OWL statement for defining ICD-10-CM categories, the percentage of Dione classes with axioms is 93,3 % (with an average of 4,8 axioms per class of the disease hierarchy, *affects* being the most used Dione object property as shown in Table 2). Dione currently contains 391,669 classes; 21,616 classes (5,5 %) taken from the ICD-10-CM hierarchy and 370,053 classes (94,5 %) taken from the SNOMED CT imported hierarchies. As we have mentioned in the Methods section, this version of Dione is still incomplete, and we could still obtain incorrect inferences from the reasoner, for example, when we don’t have a definition for a specific class. In addition, in some cases we could not find an OWL statement to distinguish between subclasses. Therefore, some classes are inferred to be equivalent to their descendants. Figure 7 presents an extract of Dione which shows how Dione looks when classes have complete definitions. In this example, A00 (Cholera) has two subclasses A00_0 (Cholera due to Vibrio cholerae 01, biovar cholerae) and A00_1 (Cholera due to Vibrio cholerae 01, biovar eltor). The definition of A00 is included in the definition of its subclasses because they are both Cholera. However, the definition of A00_0 and A00_1 includes a new owl:someValuesOf statement to distinguish between the different types of Cholera. If we are to solve all the incorrect inferences we will have to find new SNOMED CT/ICD-10-CM mappings as well as align Dione with other medical terminologies and/or ontologies so as to complete the definition of the classes. It may also be possible to obtain information from new mappings established and reviewed by the scientific community (UMLS and Bioportal) or from an expert (i.e. doctor) to complete the definition of some classes.

### Validation of Dione axioms

The Dione axioms have been included using the SNOMED CT/ICD-10-CM mappings, which has been constructed by a collaborative community of trained terminology specialists (closely following the methodology of SNOMED CT to ICD-10 Crossmap project). These final mappings are published only if they have been established as identical by a group of experts and pass a final review. In the case that SNOMED CT/ICD-10-CM mappings are not available for certain ICD-10-CM category, BioPortal provides SNOMED CT/ICD-10-CM mappings that have been previously inferred by the BioPortal algorithm and/or included (and validated) by the BioPortal user community [33, 34]. The SNOMED CT/ICD-10-CM mappings that are equal to the UMLS mappings have been removed to avoid duplicate ICD-10-CM inclusions.

As the percentage of Dione classes with axioms is 93,3 %, it is worth noting that we could have manually completed the mappings to generate the axioms for defining those classes which do not have any axiom, either inherited from parent classes or defined. However, we prefer to release the first version of Dione using only those mappings that are available and widely accepted by the scientific community. We have called this current version Dione V0.933. As new mappings are created, new axioms will be used to complete the current version of Dione.

### Applicability of Dione in clinical use cases

As Dione is logically consistent, we have used it together with the ELK reasoner to classify clinical records. Clinical record information is codified by means of the Dione object property assertions which use SNOMED CT concepts. The objective of these use cases is to show how Dione can assist health specialists by providing ICD-10-CM classified information. We have chosen three clinical records from the Virgen de la Victoria Hospital (Málaga, Spain).

**Fig. 8:**
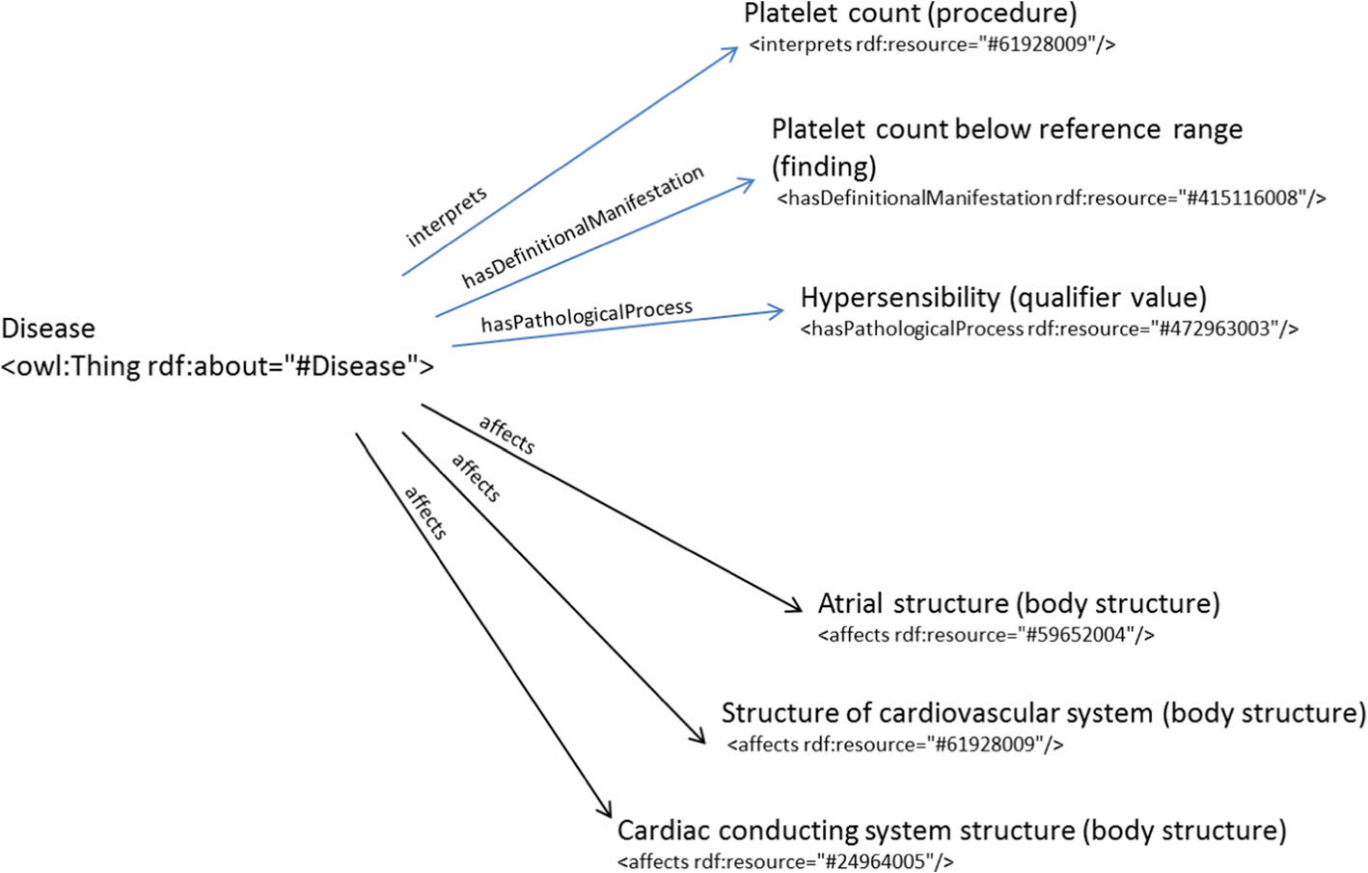
Representation of the object property assertions for the first use case. Blue and black arrows represent the object property expressions that relate individual “Disease” to the rest of individuals that correspond to SNOMED CT codes which have been manually annotated from the hematologic and main diagnoses

The first clinical record from the Hematology department describes the case of a 61-year-old man, heavy smoker, who presented with obesity, no fever, severe hypoventilation, arrhythmia and signs of hypersensibility. The results of the blood test show a low level of platelets and the electrocardiogram (ECG) determines an auricular fibrillation. The hematologic and main diagnoses are thrombocytopenia and atrial fibrillation, respectively. According to ICD-10-CM guidelines, the hematologic diagnosis code D69.6 corresponds to “Thrombocytopenia, unspecified” which was diagnosed by the health specialist. The ICD-10-CM category that corresponds to the main diagnosis is I48. For the hematologic diagnosis performed by the reasoner, instances with object property assertions such as *interprets* “Platelet count”, *hasDefintionalManifestation* “Platelet count below reference range (finding)” and *hasPathologicalProcess* “Hypersensitivity process (qualifier value)” were created and included in Dione (Fig. 8). The ELK reasoner classified the information in the Dione D69_6 class, which corresponds to the correct ICD-10-CM category. In the case of the main diagnosis, we have included object property assertions such as *affects* “Atrial structure (body structure)”, *affects* “Cardiac conducting system structure (body structure)” and “Structure of cardiovascular system (body structure)”. All this information was classified in the I48 class, which corresponds to the ICD-10-CM category of “Atrial fibrillation and flutter”. The list of inferred classes provided by the reasoner also contains other diseases that involve atrial dysfunction because the lack of definitions to distinguish between classes in the same ICD-10-CM chapter and few ones belonging to a different chapter, because the lack of class definitions (see “Level of completion of Dione” sub-section). This first use case is an example of how information from two complementary diagnoses of a given patient can be included in Dione and reasoned by ELK.

**Fig. 9:**
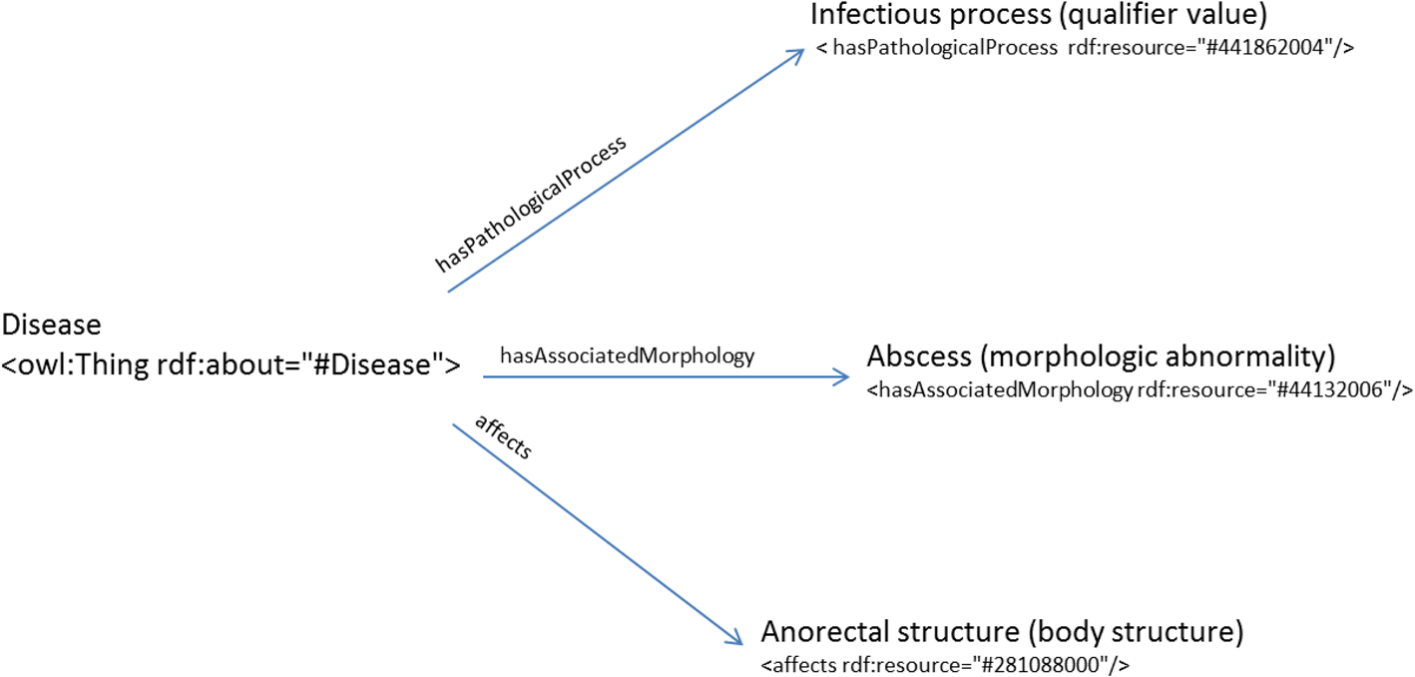
Representation of the object property assertions for the second use case. The black arrows represent the object property expressions that relate individual “Disease” to the rest of individuals that correspond to SNOMED-CT codes. The SNOMED CT codes have been manually annotated from the medical report

The second clinical record from the department of General Clinical Surgery and Digestive Apparatus describes a 53-year-old man who presented with pain, no fever and swelling of the perianal area that is identified by the clinician as an abscess. The blood test revealed a high level of leukocytes. The main diagnosis was an “Ischiorectal abscess” that corresponds to the K61.3 class. The object property assertions to be included in Dione and classified by the ELK reasoner are: *affects* “Anorectal structure (body structure)”, *hasAssociatedMorphology* “Abscess (morphologic abnormality)” and *hasPathologicalProcess* “Infectious process (qualifier value)” (Fig. 9). The reasoner classified this information in the ICD-10-CM K61 class, which corresponds to the “Abscess of anal and rectal regions”. This class includes a further five classes: K61.0 (Anal abscess), K61.1 (Rectal abscess), K61.2 (Anorectal abscess), K61.3 (Ischiorectal abscess) and K61.4 (Intrasphincteric abscess). Our objective is to provide the health specialist with an as accurate as possible ICD-10-CM code taken into account the information obtained from the EHR. This use case is an example of how additional annotations by the health specialist on the patient’s clinical record can lead to a more exact diagnosis with the proposed approach. For example, if the clinician had annotated “Ischiorectal fossa structure” on the clinical record, an additional individual with the object property assertions *affects* some “Ischiorectal fossa structure (body structure)” would have been included in Dione and therefore, the information would have been classified in a more specific ICD-10-CM class, like K61.3.

**Fig. 10:**
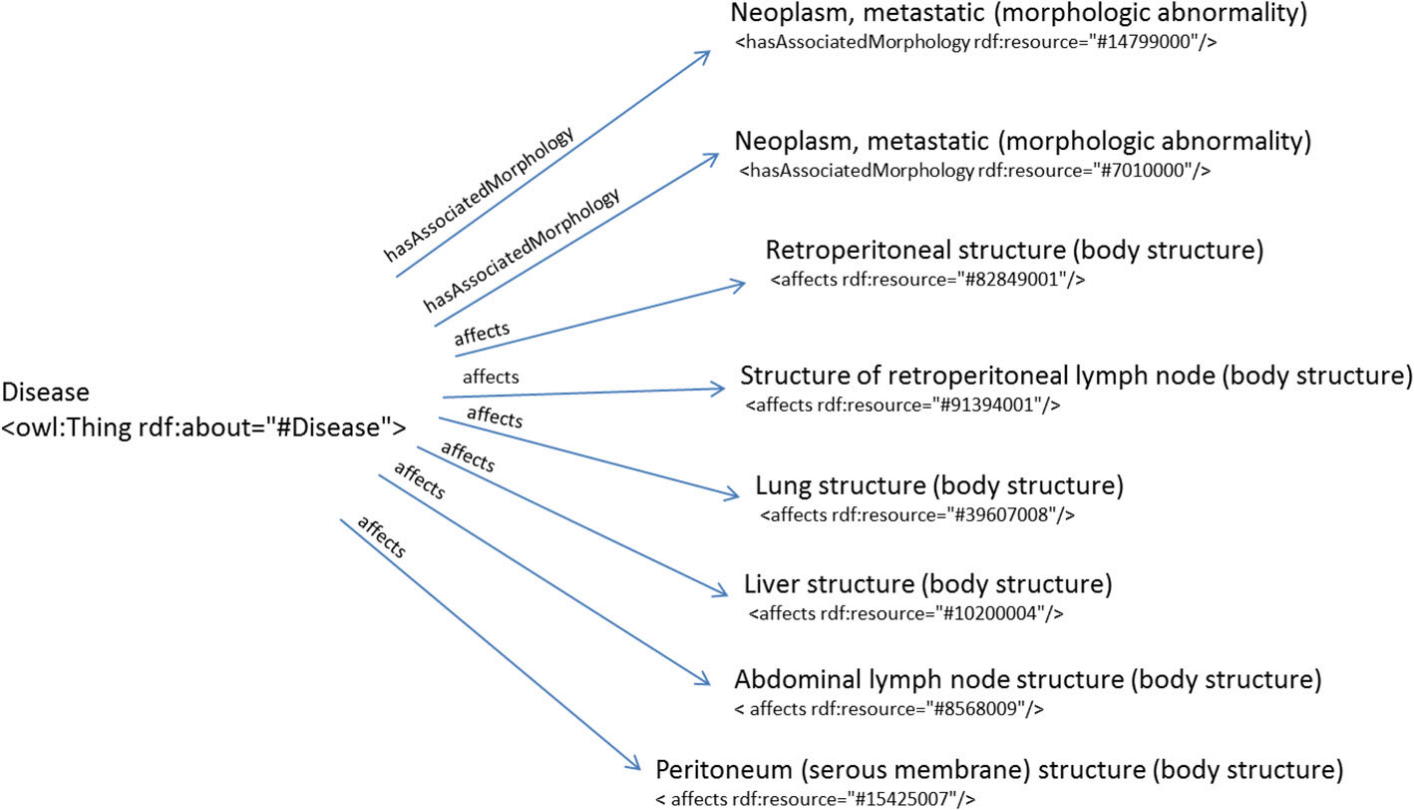
Representation of the object property assertions for the third use case. The black arrows represent the object property expressions that relate individual “Disease” to the rest of individuals that correspond to SNOMED CT codes. The SNOMED CT codes have been manually annotated from the medical report

The third clinical record from the department of Internal Medicine and Nephrology describes the case of a 77-year-old woman who underwent surgery to remove renal carcinoma. The patient did not undergo chemotherapy treatment and received the usual treatment with Bisoprolol and acetylsalicylic acid. For the next few months following the surgery, she lost weight. The clinician’s exploration through an abdominal computerised tomography revealed several neoplasms in lung, liver, in the retroperitoneal space and also some retroperitoneal adenopathies. The diagnosis was multiple neoplasms that had spread from the primary renal cancer. The diagnosis involves several ICD-10-CM categories, which are included in the class C78 (“Secondary malignant neoplasm of respiratory and digestive organs”) such as C78.0 (“Secondary malignant neoplasm of lung”), C78.3 (“Secondary malignant neoplasm of other and unspecified respiratory organs”) and C78.80 (“Secondary malignant neoplasm of unspecified digestive organ”). The object property assertions that have been defined are *hasAssociatedMorphology* “Neoplasm metastasic (morphologic abnormality)”, *affects* “Structure of retroperitoneal lymph node (body structure)”, “Lung structure (body structure)”, “Liver structure (body structure)”, “Peritoneum (serous membrane) structure (body structure)”, “Abdominal lymph node structure (body structure)” and “Retroperitoneal structure (body structure)” (Fig. 10). The disease instance was classified in the following ICD-10-CM codes: C78, C78.30 (“Secondary malignant neoplasm of unspecified respiratory organ”) and C78.80. This third use case describes a second diagnosis of a patient who had originally suffered from renal cell carcinoma. The diagnosis by the health specialist involves several ICD-10-CM categories and the information extracted from the clinical record includes information from different body structures. Therefore, according to the results obtained in this use case and the others, Dione has been able to provide a list of classified instances to help doctors classify clinical information from medical records.

### Advantages of formalising the ICD-10-CM categories in OWL

Two possible approaches can be applied to formally define the ICD-10-CM disease terms: 1) build an OWL representation to be reasoned by the OWL reasoners, as proposed in this paper and 2) establish mappings between ICD-10-CM with other terminologies and ontologies and thereby, apply indirect reasoning [35]. This second approach has been adopted by UMLS with the SNOMED CT to ICD-10-CM map. This map has been applied in the I-MAGIC algorithm [36], a Java program in which these mappings are applied using the mapping rules. The BioPortal website has also adopted the same methodology and provides mappings between ICD-10-CM terms and other ontologies. However, it is has to be noted that this second approach has some drawbacks:

1. There are two types of SNOMED CT/ICD-10-CM mappings: one-to-one and one-to-many mappings. This means that not every SNOMED CT concept can be mapped to only one ICD-10-CM categories with an identical meaning. Rather it can be mapped to more than one ICD-10-CM categories with several meanings.
2. This approach does not allow direct reasoning based on hierarchy relationship of ICD-10-CM categories established by *owl:subClassOf* axiom, the axioms included in *owl:equivalentClass* axioms to define the ICD-10-CM categories and the type of object properties that are established in the OWL model.

For these reasons, we have built an OWL hierarchy with ICD-10-CM categories and used the SNOMED CT/ICD-10-CM mappings to model the ICD-10-CM inclusion terms. According to the applicability of Dione to real clinical use cases demonstrated in the Results section, this approach provides users with a direct OWL reasoning over a set of instances from one or more actual problems (Electronic Health Records) proposed by the physician to infer new relationships and provide a new approach to the classification problem.

### Comparison with other OWL ICD models

The representation from a clinical terminology to an OWL model can cause semantic inconsistencies. According to the reviewed literature (Background section), there is a lack of consistency checking in the proposed ICD-10-CM formal representations and therefore, ABox and TBox classifications^3^ have not been done. In this approach, Dione has been validated by the ELK reasoner, which was found to be the only reasoner able to classify it, after testing all reasoners that have been successful in classifying large and widely-used real-world ontologies like SNOMED CT [37]. The ELK reasoner returns that Dione is consistent and performs TBox and ABox classifications. In order to carry out the ABox classification of Dione, a set of instances from clinical use cases taken from the Virgen de la Victoria Hospital (Málaga, Spain) has been included in Dione and classified to ICD-10-CM categories as is fully explained in the Results section.

In the Background section, we highlighted some limitations of existing work in the literature. In this section, we discuss the formal representation in OWL of ICD-10-CM proposed by [19] given that it is an ICD-10-CM representation that has improved upon other approaches. In this approach, the authors created an ICD-10-CM hierarchy with *owl:subClassOf* handling the ICD-10-CM exclusions with *owl:disjointWith* axioms. We consider that such an approach is limited given that only using *owl:disjointWith* could result in a lack of information, because if the same diseases is classified in two disjoint classes, the reasoner infers that the ontology is inconsistent, being unable to distinguish the correct class. Furthermore, the exclusions that are shared with multiple exclusions are modelled with OWL-Full, making it impossible to validate and classify the ontology with a reasoner. The inclusion terms are modelled in the same way as the exclusions. In our case, the semantic Disease hierarchy of Dione is constructed with *owl:subClassOf* axioms (using the ICD-10-CM concepts which have not been used in other approaches in the literature) and the inclusion terms are modelled from the information extracted from SNOMED CT/ICD-10-CM mappings. The approach adopted solves the problem of integrating features from OWL-Full. As mentioned, in the case of dealing with the exclusions, the mappings include an exhaustive mapping of the low-level descendants of those SNOMED CT concepts that could lead to a different ICD-10-CM category given ICD-10-CM exclusions and other rules.

## Conclusions

This paper has presented the implementation process of Dione, an OWL representation of ICD-10-CM, which uses SNOMED CT/ICD-10-CM mappings to formalise the ICD-10-CM diseases categories and their inclusion terms. The main hypothesis guiding us is: **(H1) It is possible to code, as OWL axioms, the ICD-10-CM inclusion terms obtained from SNOMED CT/ICD-10-CM mappings and use these OWL axioms to build an OWL representation of the ICD-10-CM diseases**. Therefore, we have used an automatic process to build a hierarchy tree with ICD-10-CM disease categories and their axioms using the *owl:equivalentClass* axiom. The main objective of our approach has been to build a model that can be used by a reasoner. Therefore, we have also shown that Dione is consistent and a TBox classification has been carried out by the ELK reasoner. It is worth noting that the automatisation of the ICD-10-CM disease categories is important given that new mappings are continuously being added to complete Dione, whose initial version is released with this paper. In its current version, we have not been able to find validated SNOMED CT/ICD-10-CM mappings for each ICD-10-CM category and so did not have correct results in several cases. Therefore, our first objective is to complete the definition of all classes. We plan to add more mappings from other ontologies which will relate Dione with more biological information from different areas that involve personalised medicine. BioPortal provides ontologies mapped to ICD-10-CM categories such as the National Drug Data File (with 2,857 ICD-10-CM mappings) [38], OMIM (with 3,921 ICD-10-CM mappings) [39], the Human Phenotype Ontology (with 1,370 ICD-10-CM mappings) [40] and the Regulation of Transcription Ontology (with 61 ICD-10-CM mappings) [41, 42]. Using the approach presented in this paper, the mappings can be extracted from BioPortal and stored in a database. The axioms to be included in Dione using *owl:equivalentClass* can be defined with an “affect” object property or also with the axioms that are defined in the ICD-10-CM mapped class of the target ontology. It may also be possible to obtain information from an expert (i.e. doctor) to complete the definition of some classes.

As a secondary hypothesis, we **(H2) obtain a useful OWL representation which can be used as the basis for a semantic classification system**. This enables a set of SNOMED CT concepts and their relationships, taken from EHRs, as input to automatically classify patients’ diseases into an ICD-10-CM category. This has been tested by including object property assertions in Dione from the Virgen de la Victoria Hospital’s (Málaga, Spain) clinical records which have been classified into ICD-10-CM categories showing the applicability of the OWL representation. After completing Dione, we plan to measure the accuracy of the classification and to study new ways to improve it. As the development of Dione is ongoing, further work will include looking at how Dione reasoning can assist experts in providing classified information for ICD-10-CM disease categories from actual use cases of patient records. Dione should be supported in the future by a semi-automatic EHR annotation tool, probably based on text mining and natural language processing techniques. Our intention is to use an end-user interface where the information processed can be displayed in such a way as to make it easily understandable to specialists in the field. This application could provide functionalities which allow users to make specific OWL queries such as: *“Find a disease which affects a body structure (finding site) like “Systematic circulatory system structure (body structure)” AND hasDefinitionManifestation “Finding of increase blood pressure (finding)”*. These kinds of queries can be used to retrieve an ICD-10-CM disease category that has the same definition in Dione and also, to automatically generate a list of possible ICD-10-CM categories in which the instances from real clinical records can be classified. Finally, we will study how Dione could be combined with other techniques such as SWRL (Semantic Web Rule Language) rules [43] and probabilistic databases in order to develop a diagnostic assistance tool.

## Endnotes

^1^Dione is available at http://www.khaos.uma.es/dione

^2^According to the semantics of OWL, this represents an anonymous class. It has an object property **hasDefinitionalManifestation**. At least one value for hasDefinitionalManifestation must be an instance of 24184005 “Finding of increased blood pressure (finding).

^3^TBox and ABox are known as terminological and assertion components, respectively. TBox statements describe the set of Dione classes and properties and ABox statements are used to describe the instances associated with those classes and properties.

## Additional files

Additional file 1 Creating the ICD-10-CM categories tree. PDF file containing the algorithm for parsing the XML-file containing the ICD-10-CM categories to output a tree with parent and child nodes. (PDF 82 kb)

Additional file 2 Representing ICD-10-CM categories in OWL. PDF file containing the algorithm for creating the ICD-10-CM representation in OWL. (PDF 73 kb)

Additional file 3 Including OWL axioms in Dione. PDF file containing the algorithm for obtaining axioms from SNOMED CT/ICD-10-CM mappings and for adding these axioms to Dione. (PDF 73 kb)

Additional file 4 Imported hierarchies. PDF file containing the algorithm for creating SNOMED CT imported hierarchies and for including these hierarchies in Dione. (PDF 73 kb)

Additional file 5 Dione level of completeness. PDF file containing the algorithm for recursively counting the number of classes defined using relationships from the SNOMED CT/ICD-10-CM mappings. (PDF 72 kb)

Additional file 6 Classification of Dione. PDF file containing the algorithm for including the axioms defining a class in the definition of its subclasses. (PDF 68 kb)

Additional file 7 Classification of Dione. PDF file containing the algorithm for including the owl:someValuesFrom statement from the definition of sibling subclasses in the definition of their superclass. (PDF 68 kb)

Additional file 8 Classification of Dione. PDF file containing the algorithm for classifying Dione using the ELK reasoner through the OWL API. (PDF 72 kb)

## Abbreviations

CDC: Center for disease control and prevention
CRM: Core reference model
DRGs: Diagnosis-related groups
EHR: Electronic health record
FMA: Foundational model of anatomy
ICD-10: International classification of diseases, tenth revision
ICD-10-CM: International classification of diseases, tenth revision, clinical modification
IUPAC: International union of pure and applied chemistry
NIH: National institutes of health OWL: Web ontology language
SNOMED CT: Systematized nomenclature of medicine - clinical terms
UMLS: Unified medical language system
WHO: World health organisation
