# Supplementary material for: Dione: An OWL representation of ICD-10-CM for classifying patients’ diseases
Source: J Biomed Semantics. 2016 Oct 13;7:62. doi: 10.1186/s13326-016-0105-x (PMC5064922; doi:10.1186/s13326-016-0105-x)
Supplement: Additional file 1 — Creating the ICD-10-CM categories tree. PDF file containing the algorithm for parsing the XML-file containing the ICD-10-CM categories to output a tree with parent and child nodes. (PDF 82 kb) [file 13326_2016_105_MOESM1_ESM.pdf]

Algorithm 1 Create a file with ICD-10-CM hierarchy from "ICD10CM_FY2014_Full_XML_Tabular.xml" file

```
procedure Create a file with ICD10-CM-10 hierarchy
    root = getRootNode("ICD10CM_FY2014_Full_XML_Tabular.xml");
    NodeList sections = getChilds(root, "section");
    for each node n from sections do
        section = getItem(i);
        printSection(section)
    end for
    function printSection(section)
        id = getNodeAttribute(section, "id");
        desc = getChildText("desc");
        printFile(id + des);
        printFile(id + "Diseases" + des);
        NodeList sections = section.getChildNodes();
        if sections!=null then
            for each node n from sections do
                section = getItem(n);
                if section.equals("diag") then
                    printDiag(section, 1, id);
                end if
            end for
        end if
    end function
    function printDiag(section, tabs, father )
        id = getChildText("name");
        des = getChildText("desc");
        for each iteation i create a tabs do
            print(tabs);
        end for
        printFile(res + des);
        printFile(id + padre + des);
        NodeList diags = getChildNodes();
        if diags!=null then
            for each node n from diags do
                diags.item(n);
                if section.equals("diag") then
                    printDiag(diag, tabs+1, id);
                end if
            end for
        end if
    end function
end procedure
```
