# Supplementary material for: Dione: An OWL representation of ICD-10-CM for classifying patients’ diseases
Source: J Biomed Semantics. 2016 Oct 13;7:62. doi: 10.1186/s13326-016-0105-x (PMC5064922; doi:10.1186/s13326-016-0105-x)
Supplement: Additional file 2 — Representing ICD-10-CM categories in OWL. PDF file containing the algorithm for creating the ICD-10-CM representation in OWL. (PDF 73 kb) [file 13326_2016_105_MOESM2_ESM.pdf]

Algorithm 2 Creation of the ICD-10-CM representation in OWL

```
procedure ICD-10-CM REPRESENTATION IN OWL
    manager = createOWLOntologyManager();
    ontologyIRI = createIRI
    ("http://www.semanticweb.org/ontologies/2013/11/icd10k.owl#");
    factory = manager.getOWLDataFactory();
    ont = manager.createOntology(ontologyIRI);
    readLine();
    while line (from ICD-10-CM tabulator tree file) !=null do
        get child class;
        getOWLClass(child);
        getOWLClass(parent);
        getLabel(label);
        addAxiom(getOWLSubClassOfAxiom(child, parent));
        getOWLAnnotation(getRDFSComment(), getOWLLiteral(label))
        getOWLAnnotationAssertionAxiom();
    end while
    saveOntology(ont);
end procedure
```
