# Supplementary material for: Dione: An OWL representation of ICD-10-CM for classifying patients’ diseases
Source: J Biomed Semantics. 2016 Oct 13;7:62. doi: 10.1186/s13326-016-0105-x (PMC5064922; doi:10.1186/s13326-016-0105-x)
Supplement: Additional file 3 — Including OWL axioms in Dione. PDF file containing the algorithm for obtaining axioms from SNOMED CT/ICD-10-CM mappings and for adding these axioms to Dione. (PDF 73 kb) [file 13326_2016_105_MOESM3_ESM.pdf]

Algorithm 3 Get axioms from ICD-10-CM/SNOMED CT mappings and addition to the DIONE ontology

```
procedure GET AXIOMS FROM MAPPINGS AND ADDITION TO ONTOLOGY
    Set database connection;
    readFile(ICD-10-CM classes);
    printFileWithAxioms();
    while line (line = readLine() !=null do
        result = select ICD10, SNOMEDCT, concept1, relationshiptype, concept2 from mappings, relations where ICD10 = line and SNOMEDCT = concept1;
        while  row != null do
            get(ICD-10));
            get(SNOMEDCT));
            get(concept1);
            get(relationshiptype);
            get(concept2);
            nextline();
        end while
    end while
    addAxioms(file);
    closeFileWithAxioms();
    close connection;
    function ADDAXIOMS(FILEWITHAXIOMS)
        getInstance();
        loadOntologyWithoutaxioms();
        readAxiomsFromFile();
        createOntologyWithAxioms();
        saveOntology();
        close database connection;
    end function
end procedure
```
