# Supplementary material for: Dione: An OWL representation of ICD-10-CM for classifying patients’ diseases
Source: J Biomed Semantics. 2016 Oct 13;7:62. doi: 10.1186/s13326-016-0105-x (PMC5064922; doi:10.1186/s13326-016-0105-x)
Supplement: Additional file 4 — Imported hierarchies. PDF file containing the algorithm for creating SNOMED CT imported hierarchies and for including these hierarchies in Dione. (PDF 73 kb) [file 13326_2016_105_MOESM4_ESM.pdf]

Algorithm 4 Create imported OWL SNOMED classes from database

```
procedure CREATE IMPORTED OWL SNOMED CLASSES
    ontologyCreation(table, root);
    createOWLOntologyManager();
    ontologyIRI = createIRI("http://www.w3.org/2003/01/SNOMEDCT.owl#");
    manager.createOntology(ontologyIRI);
    manager.getOWLDataFactory();
    set database connection;
    result = select conceptid1, conceptid2, name from table;
    resultset = executeQuery(query);
    while resultset != null do
        child = getString(concept1);
        father = getString(concept2);
        name = getName(name);
        writeHierarchyInOntology(child, father, name);
    end while
    close database connection;
    function WRITEHIERARCHYINONTOLOGY(CHILD, FATHER, NAME)
        getOWLClass(IRI.create(child);
        getOWLClass(IRI.create(father);
        addAxiom(getOWLSubClassOfAxiom(child, parent))
        getOWLAnnotation(getRDFSComment(), getOWLLiteral(name))
        getOWLAnnotationAssertionAxiom();
    end function
    saveOntology();
end procedure
```
