# Supplementary material for: Dione: An OWL representation of ICD-10-CM for classifying patients’ diseases
Source: J Biomed Semantics. 2016 Oct 13;7:62. doi: 10.1186/s13326-016-0105-x (PMC5064922; doi:10.1186/s13326-016-0105-x)
Supplement: Additional file 5 — Dione level of completeness. PDF file containing the algorithm for recursively counting the number of classes defined using relationships from the SNOMED CT/ICD-10-CM mappings. (PDF 72 kb) [file 13326_2016_105_MOESM5_ESM.pdf]

Algorithm 5 Count classes without inherited/non-inherited axiom(s)

```
function checkEquivalence(class, ontology, PrintWriter axioms_ok, PrintWriter axioms_no_ok)
    PrintWriter axioms_ok;
    PrintWriter axioms_no_ok;
    ontology = loadOntologyFromOntologyDocument(ontology);
    ontologyIRI =
    create("http://www.semanticweb.org/ontologies/2013/11/icd10k.owl#");
    factory = manager.getOWLDataFactory();
    factory.getOWLClass(IRI.create(ontologyIRI + "Diseases"));
    Set subclasses = claseDiseases.getSubClasses(ontology);
    for each subclass from subclasses do
        size = subclase.asOWLClass().getEquivalentClasses(ontology).size();
        if size == 0 then
            axioms_no_ok.println(subclass.asOWLClass())
        else
            axioms_ok.println(subclass.asOWLClass())
            checkEquivalence(subclass.asOWLClass(), ontology, axioms_ok, axioms_no_ok);
        end if
    end for
end function
```
