# Supplementary material for: Dione: An OWL representation of ICD-10-CM for classifying patients’ diseases
Source: J Biomed Semantics. 2016 Oct 13;7:62. doi: 10.1186/s13326-016-0105-x (PMC5064922; doi:10.1186/s13326-016-0105-x)
Supplement: Additional file 6 — Classification of Dione. PDF file containing the algorithm for including the axioms defining a class in the definition of its subclasses. (PDF 68 kb) [file 13326_2016_105_MOESM6_ESM.pdf]

Algorithm 7 Inheritance from patent class axioms to son classes

```
procedure Get Parent class
    loadontology(ontology);
    class parent = getSuperClass();
    getSubclasses(parent);
    function getSubclasses(parent)
        List equivalentClasses = getEquivalentClass(parent);
        Iterator subClasses = getSubclasses(parent);
        while subClasses.hasNext() do
            create List empty AxiomsList;
            List equivalentClassList = getEquivalentClass(subClasses);
            while equivalentClassList.hasNext() do
                if equivalentClass is intersectionOf then
                    addToAxiomList(decomposeIntersectionAxioms(equivalentClass));
                end if
                if equivalentClass is not intersectionOf then
                    addToAxiomList(equivalentClass);
                end if
            end while
            if addToAxiomList != null then
                subClasses.addEquivalentClassList.createIntersection(addToAxiomList);
            end if
            getSubclasses(parent)
        end while
    end function
end procedure
```
