# Supplementary material for: Dione: An OWL representation of ICD-10-CM for classifying patients’ diseases
Source: J Biomed Semantics. 2016 Oct 13;7:62. doi: 10.1186/s13326-016-0105-x (PMC5064922; doi:10.1186/s13326-016-0105-x)
Supplement: Additional file 7 — Classification of Dione. PDF file containing the algorithm for including the owl:someValuesFrom statement from the definition of sibling subclasses in the definition of their superclass. (PDF 68 kb) [file 13326_2016_105_MOESM7_ESM.pdf]

Algorithm 8 Get common axioms from son classes and addition to parent classes

```
procedure GET COMMON AXIOMS FROM SONS AND ADD TO PARENT CLASSES
    loadontology(ontology);
    class parent = getSuperClass();
    getAllEquivalentClasses(parent);
    function GETALLEQUIVALENTCLASSES(PARENT)
        List equivalentClasses = getEquivalentClass(parent);
        Iterator subClasses = getSubclasses(parent);
        while subClasses.hasNext() do
            getAllEquivalentClasses(subClasses)
            Set equivalentClassSons = getAllEquivalentClasses(subClasses);
            Set elementsFromIntersection;
            Set equivalentClassSons2;
            while (equivalentClassSons.hasNext() do
                if equivalentClassSons is intersectionOf then
                    elementsFromIntersection(decomposeIntersectionAxioms(equivalentClass));
                end if
                if equivalentClassSons is not intersectionOf then
                    elementsFromIntersection(equivalentClass);
                end if
            end while
            if equivalentClassSons2 == null then
                equivalentClassSons2 = elementsFromIntersection;
            end if
            if equivalentClassSons2 != null then
                equivalentClassSons2 = findCommonAxioms(equivalentClassSons2, elementsFromIntersection);
            end if
        end while
        if equivalentClassSons2 != null then
            addEquivalentClasses(parent,equivalentClassSons2);
        end if
    end function
end procedure
```
