# Supplementary material for: Dione: An OWL representation of ICD-10-CM for classifying patients’ diseases
Source: J Biomed Semantics. 2016 Oct 13;7:62. doi: 10.1186/s13326-016-0105-x (PMC5064922; doi:10.1186/s13326-016-0105-x)
Supplement: Additional file 8 — Classification of Dione. PDF file containing the algorithm for classifying Dione using the ELK reasoner through the OWL API. (PDF 72 kb) [file 13326_2016_105_MOESM8_ESM.pdf]

Algorithm 6 Use of the ELK reasoner through OWL API to make ABox and TBox classifications

```
procedure Reasoning with ELK
    loadontology(ontology);
    createReasoner(ontology);
    precomputeInferences(InferenceType.CLASS_HIERARCHY);
    precomputeInferences(InferenceType.CLASS_ASSERTIONS);
    precomputeInferences(InferenceType.DATA_PROPERTY_ASSERTIONS);
    precomputeInferences(InferenceType.DIFFERENT_INDIVIDUALS);
    precomputeInferences(InferenceType.SAME_INDIVIDUAL);
    add(new InferredSubClassAxiomGenerator());;
    add(new InferredEquivalentClassAxiomGenerator());
    add(new InferredClassAssertionAxiomGenerator());
    saveOntology(ont);
    closeReasoner;
end procedure
```
